# Structural Basis of the Bivalency of the TRPV1 Agonist DkTx

**DOI:** 10.1002/anie.202314621

**Published:** 2023-12-12

**Authors:** Venkatraman Ramanujam, Theo Crawford, Ben Cristofori‐Armstrong, Jennifer R. Deuis, Xinying Jia, Michael J. Maxwell, Sina Jami, Linlin Ma, Irina Vetter, Mehdi Mobli

**Affiliations:** ^1^ Australian Institute for Bioengineering and Nanotechnology The University of Queensland St Lucia 4072 Queensland Australia; ^2^ Institute for Molecular Biosciences School of Pharmacy The University of Queensland St Lucia 4072 Queensland Australia; ^3^ Griffith Institute for Drug Discovery School of Environment and Science Griffith University Nathan 4111 Queensland Australia

**Keywords:** Bivalent, Disulfide-Rich Peptides, DkTx, Intein-Mediated Ligation, NMR Spectroscopy

## Abstract

Bivalency is a prevalent natural mechanism to enhance receptor avidity. Various two‐domain disulfide‐rich peptides exhibiting bivalent action have been identified from animal venoms. A unique characteristic of these peptides is that they induce a pharmacological response different from that provoked by any of the constituent domains. The enhanced potency and avidity of such peptides is therefore a consequence of their domain fusion by a peptide linker. The role of the linker itself, beyond conjugation, remains unclear. Here, we investigate how the linker affects the bivalency of the capsaicin receptor (TRPV1) agonist DkTx. We recombinantly produced isotope labelled DkTx using a protein splicing approach, to solve the high‐resolution solution structure of DkTx, revealing residual linker order stabilised by linker‐domain interactions leading to biased domain orientations. The significance of this was studied using a combination of mutagenesis, spin relaxation studies and electrophysiology measurements. Our results reveal that disrupting the pre‐organisation of the domains of DkTx is accompanied by reductions in potency and onset of avidity. Our findings support a model of pre‐configured two‐domain binding, in favour of the previously suggested sequential binding model. This highlights the significance of ordered elements in linker design and the natural evolution of these in bivalent toxins.

## Graphical abstract

Using protein‐splicing, we produced a labelled bivalent capsaicin receptor agonist, DkTx, for NMR characterisation. The structure reveals linker pre‐organisation stabilised by interaction between the first domain and the linker. Disrupting this reduces linker order, affecting avidity at sub‐saturating concentrations, illustrating how linker order enhances potency of bivalent receptor engagement.

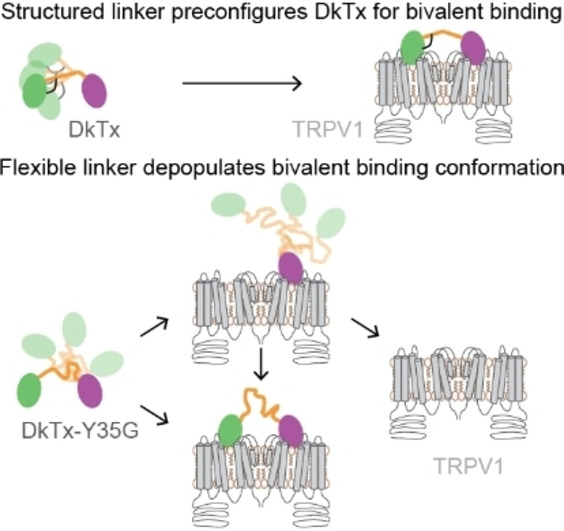

## Introduction

Bivalency is a prominent natural mechanism that augments the potency and specificity of intermolecular interactions. This mechanism accounts for the high avidity of antibodies while also being an important strategy in drug development.^[1]^

Recently, several venom peptides with bivalent properties have emerged.[^2^, ^3^] Structurally, these peptides are tandem repeats of disulfide‐stabilised domains joined by a linker. Recent analysis of available protein sequences, revealed that such peptides are members of a broader class termed “secreted cysteine‐rich repeat proteins” or SCREPs.[^4^, ^5^] The unusual bivalency of the tandem repeat spider toxin DkTx (double‐knot toxin), an agonist of the capsaicin receptor TRPV1, has been characterised in detail.^[2]^ DkTx comprises a tandem repeat of the inhibitor cysteine knot (ICK) motif. Subsequently, a second tandem repeat ICK peptide, Hi1a, from a different spider venom, was also shown to have bivalent action, this time towards acid sensing ion channel 1a (ASIC1a).^[3]^ Most recently, a tandem repeat ICK peptide, Xt3a, from the venom of a remipede was shown to be a bivalent agonist of ryanodine receptors (RyR).^[6]^ The bivalency of these peptides is remarkable, as simple conjugation of two proteins does not itself necessarily result in enhanced potency and avidity as is evident in some peptide dendrimers.^[7]^ These proteins, therefore, have evolved to simultaneously target distinct receptor sites and are connected by a linker that allows them to achieve this efficiently.

The precise role of the peptide linker in multi‐domain toxins, in relation to their bivalency, remains elusive. In the case of DkTx, the peptide‐receptor complex is known, but the resolution of the structure does not reveal structural details of the linker, and a solution structure of the tandem repeat peptide is not available.[^8^, ^9^] Conversely for Hi1a and Xt3a, the solution structure is known, and details of the inter‐domain orientation and the structure of their linkers are observable in solution, while the peptide‐channel complexes are unknown, making it difficult to conclude how the linker may affect the bivalency of these peptides.[^3^, ^6^]

In DkTx, the bivalent binding of the peptide has been proposed to follow a sequential binding model, where the linker simply acts as a tether to enhance the local concentration of the second domain, following single‐domain binding.[^9^, ^10^] In this model, the higher potency of DkTx compared to that of the most potent constituent knot (ICK) is difficult to explain, and instead suggests that both domains bind simultaneously. This two‐domain binding mode may require some level of preorganisation of the two domains directed by structural elements in the linker of DkTx.

To investigate if there is evidence of linker order and biased domain organisation, we set out to characterise the peptide structure and dynamics in solution by multidimensional heteronuclear NMR spectroscopy. Structural studies by NMR can provide high‐resolution structural details of pre‐organisation of the peptide linker in solution,[^3^, ^11^] and spin‐relaxation studies provide direct measures of the level of disorder in the inter‐domain linker.^[12]^ Such NMR studies require ^15^N and ^13^C isotope labelling. Thus, we developed an intein‐based method for in vivo ligation of the individual domains of DkTx, to improve the yield of recombinantly produced DkTx in *E. coli*. The method presents a useful alternative approach to producing tandem repeats of disulfide‐rich peptides, which can be shown to generally reduce the combinatorial problem of oxidative folding of multidomain disulfide‐rich peptides.

Our results reveal that the solution structure of DkTx displays residual order in the interdomain linker region, which we attribute to tyrosine stacking between a residue in the first domain and the linker. Disruption of this aromatic stacking leads to reduced order in the linker. Functional studies show that this leads to an increase in the concentration required for bivalent engagement of the channel. These results support a model of simultaneous binding of the two domains of DkTx in favour of sequential binding of the domains. Together, our results demonstrate an evolutionary strategy where inter‐domain linkers can evolve to lower the minimal concentration required for irreversible channel engagement of multivalent toxins. Revealing linker stabilisation as a potentially important trait in molecular evolution of animal venoms, with practical applications in contemporary drug development.

## Results and Discussion

### In vivo ligation of intein spliced iDkTx

Recombinant protein ligation can be achieved by a number of methods. A particularly attractive method for ligating two independently folded domains is through a process known as protein trans‐splicing,[^13^, ^14^] which leaves a relatively small ligation scar (insertion of non‐native amino acids).^[15]^ This ligation is catalysed by a subgroup of protein splicing elements known as inteins. In split inteins, the precursor protein is fragmented into two separate genes, with the break in sequence occurring within the intein (see Figure S1).^[13]^ The protein splicing mechanism progresses in four concerted nucleophilic displacement reactions,^[16]^ which are preceded by an association step between the N‐ and C‐terminal split inteins. The residues proximal to the splice junction and residues within the inteins direct these ligation reactions. In addition, at the first C‐extein position (+1 residue) a nucleophilic residue is required for splicing activity.^[17]^ The low p*K*_a_ of the cysteine thiol group makes this an efficient nucleophile. However, achieving independent folding of the two disulfide‐rich domains requires that there are no free cysteines present in the linker region—or equivalently that the junction is not made at the first or last cysteine residue of the ligated domains. To avoid these complications a serine at the +1 residue position can be used.

This strategy allows for independent folding of the two domains, and has theoretical appeal as it limits the number of possible disulfide isoforms, e.g. a peptide with 12 cysteines can form up to 10395 isoforms (excluding topological isoforms), but only 225 isoforms if the two domains are folded independently (15 isoforms in each 6‐cysteine domain and all their possible ligations—again excluding topological isoforms).

**Figure 1:**
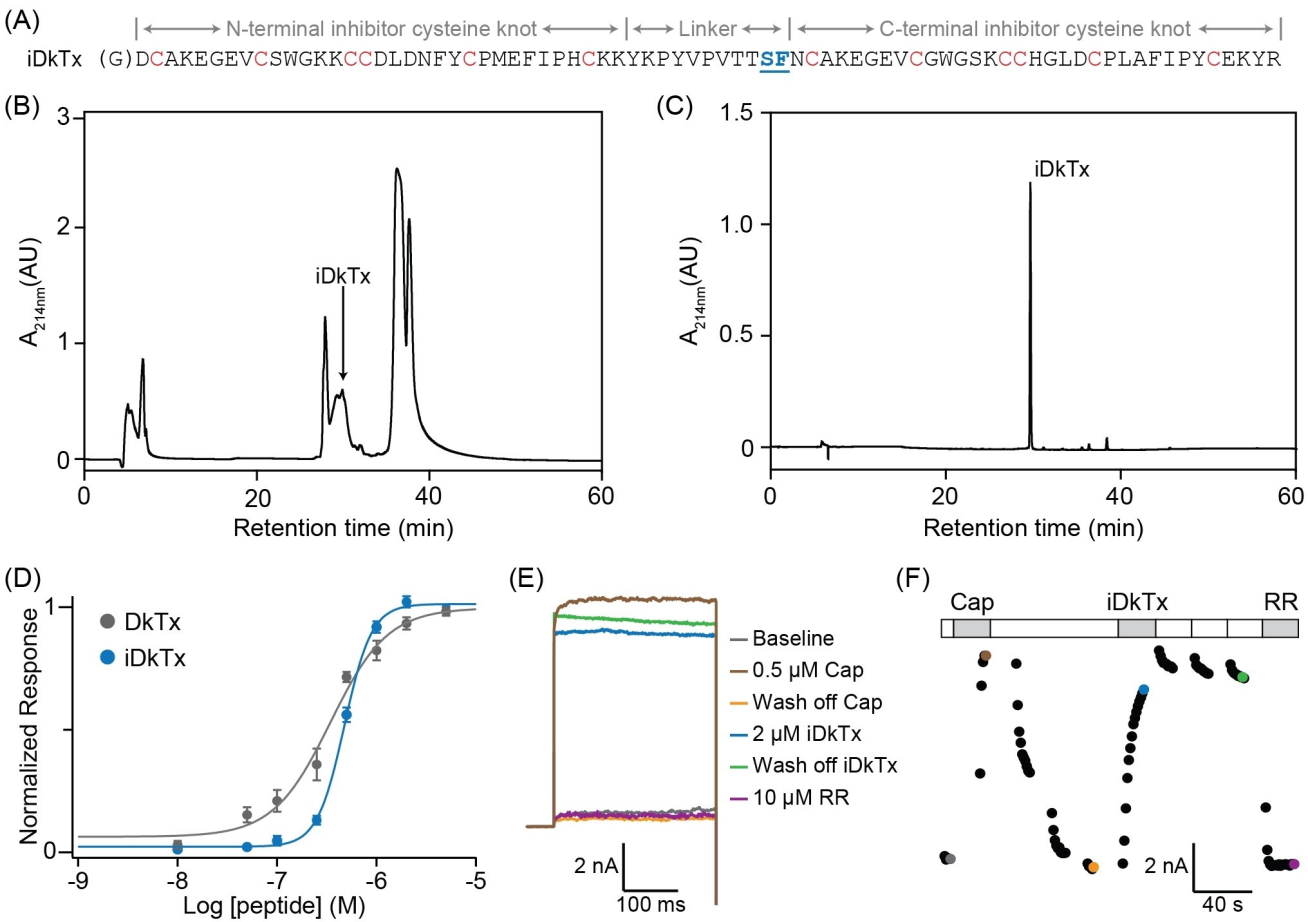
Purification and functional characterisation of in vivo, intein spliced, DkTx (iDkTx). (A) The final sequence of iDkTx after in vivo intein splicing, with the ligation scar highlighted in blue and underlined, the cysteines shown in red, and the two domains and the interdomain linker annotated in grey. The N‐terminus of iDkTx contains a non‐native glycine shown in brackets that is a vestige from the TEV protease site. (B) RP‐HPLC chromatogram of the nickel‐affinity elution fraction containing in vivo ligated iDkTx. (C) Analytical RP‐HPLC chromatogram showing pure iDkTx following cleavage to remove the His‐tag. (D) iDkTx and DkTx are equipotent as determined by calcium imaging with TRPV1‐expressing HEK293 cells. Concentration‐response analyses of iDkTx (EC_50_ 471 nM) and DkTx (EC_50_ 345 nM) with data normalised to maximal responses for each peptide. Data are mean ± standard error of the mean (*n*=4–6). (E) Whole‐cell patch‐clamp recording with TRPV1‐expressing HEK293 cells showing the maximum TRPV1 current (+80 mV) activated by 0.5 μM capsaicin (Cap) and 2 μM iDkTx, which can be inhibited by 10 μM ruthenium red (RR). (F) The time course of the experiment in (E) shows that the capsaicin response is rapidly reversible upon washing, whereas iDkTx produces persistent membrane currents that are not reversible through washing. However, they can be fully inhibited by 10 μM ruthenium red (RR). Individual data points that are coloured corresponding to the equivalent experimental trace shown in panel (E).

To utilise this protein splicing approach, we employed a split intein system where the transesterification reaction can be catalysed by a serine.^[18]^ The natural DnaE split inteins from *Nostoc punctiforme* (*Npu*) and *Synechocystis sp*. (*Ssp*) have been the ‘gold standards’ for both in vivo and in vitro protein ligation reactions because of their favourable solubility when over‐expressed.^[19]^ Based on our initial trials (Figure S1), the combination of *Npu* DnaE_N_ and *Ssp* DnaE_C_ with a catalytic serine was the most efficient trans‐splicing condition, and used for subsequent optimisation. In addition to the catalytic residue (+1 extein residue), it has been shown that the +2 extein residue is also important for protein splicing efficiency, with phenylalanine yielding the highest efficiency.^[15]^ Hence, a ligation scar consisting of only the catalytic serine (+1) and phenylalanine (+2) is retained in our final intein DkTx (henceforth iDkTx) construct (Figure 1 A).

In the iDkTx construct, the N‐terminal precursor protein contains an N‐terminal hexa‐histidine tag (His_6_), a TEV protease cleavage site and the ICK1 sequence of DkTx followed by the *Npu* DnaE_N_ intein sequence. The C‐terminal precursor protein contains the *SSp* DnaE_C_ intein sequence (ending with the splice site SF) followed by ICK2 of DkTx. Furthermore, since each split intein is under the control of a different inducible promoter (T7 and araBAD), it is possible to selectively overexpress the precursor proteins in media containing different isotopes, resulting in segmentally labelled proteins^[20]^ (Figure S2), demonstrating that the two domains are expressed and allowed to fold independently.

The sequential in vivo expression and subsequent splicing of the two precursor proteins yields the N‐terminally hexa‐histidine tagged full‐length DkTx, containing a TEV protease cleavage site between the His‐tag and the toxin sequence. Following expression, the protein is obtained in a mixture of disulfide isoforms, where the yield of the folded state is further increased using established refolding conditions during cleavage of the His‐tag by TEV protease (Figures 1 and S1). The sequence of our final iDkTx product is similar to that used by Bohlen et al.,^[2]^ where a genenase‐I protease cleavage site containing 3 amino acids (H‐Y‐R) was introduced between ICK1 and ICK2 at the same position. The HYR‐modified DkTx was found to be equipotent to the native DkTx, and indeed we find that our engineered iDkTx retains the potency and avidity of the parent compound (Figure 1D–F).

### NMR structure of iDkTx reveals linker tyrosine stacking

**Figure 2:**
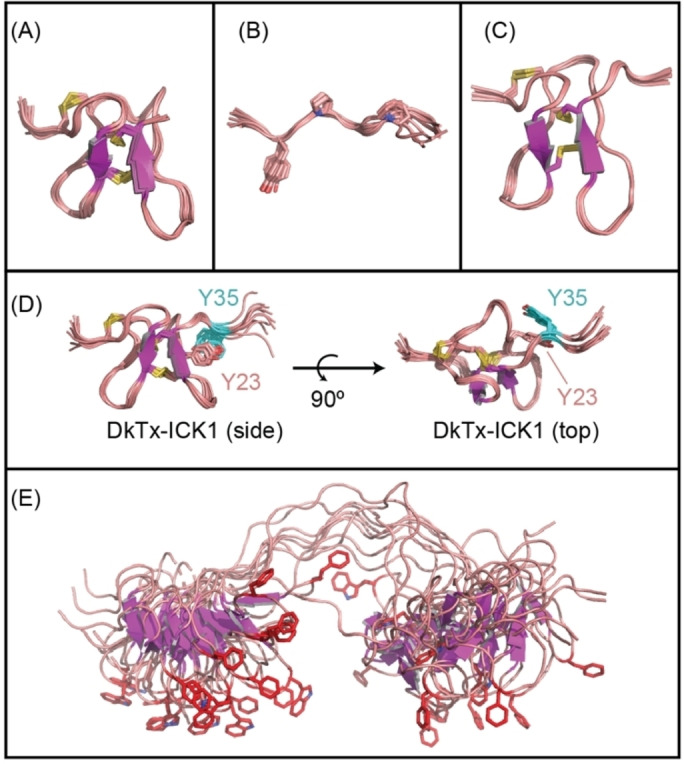
Structural characterisation of iDkTx (PDBID: 6CUC). Ensemble of top 10 structures (by target function) of: (A) ICK1, (B) linker region (residues 34–42 shown—linker prolines and Y35 shown as sticks) and (C) ICK2, with backbone atomic position RMSD of 0.35, 0.36 and 0.18 respectively. (D) Tyrosine stacking of ICK1 and linker residues (Y23 and Y35 in iDkTx; Y22 and Y34 in WT numbering), shown from top and side views. (E) Global overlay of the iDkTx ensemble, showing the result of the residual linker structure on the position of channel binding aromatic residues in each ICK (red sticks).

Next, we uniformly labelled iDkTx with ^15^N and ^13^C isotopes for structural characterisation by heteronuclear NMR spectroscopy. Near complete resonance assignment was achieved (95.8 % backbone atoms, 81.9 % of sidechain carbons and 94.8 % sidechain protons were assigned, BMRB: 27433—Figure S3). The assigned chemical shifts were used to generate dihedral angle restraints (TALOS‐N^[21]^). These restraints were supplemented with ≈1000 distance restraints from NOEs in the torsion angle dynamics software CYANA^[22]^ for structure calculations (see Figure 2 and Table S1—PDBID: 6CUC). Overall, the structure of each ICK domain is in close agreement with the published structures of the individual domains of DkTx.^[23]^

The structure of the full‐length protein allowed us to make additional observations with respect to the interdomain linker. Firstly, we find that residues near the introduced SF sequence have chemical shifts close to random coil values, indicating a high level of disorder. In particular, four amino acids [42–45, TTSF] in the C‐terminal end of the linker are the only residues that have random coil derived order parameter (S^2^) values below 0.5 (as predicted by TALOS). This is consistent with the observed minor functional consequence of the HYR or SF insertion in this region. Second, we find that the N‐terminal end of the linker retains some structural stability (reflected in low RMSDs of atomic coordinates—Figure 2).

We identified that the source of the residual structure was the formation of a stable aromatic stack between Y35 in the linker and Y23 in ICK1 (Figure 2‐D; Y34 and Y22 in WT‐DkTx). This aromatic stacking interaction restricts the conformational space that can be sampled by the linker region. Importantly, in the structural ensemble there is a preference for alignment of the two domains in an orientation where the loop regions containing the important channel binding residues [W12 and F28 in ICK1 and W56 and F70 in ICK2 of iDkTx (W11, F27, W53 and F67 in WT‐DkTx)] point in the same direction (Figure 2‐E), further indicating presence of restricted motion in the linker, which may underlie the avidity of the peptide.

### Tyrosine stacking stabilises the linker in DkTx

To further probe the role of the observed ICK1‐linker interaction in pre‐organising the structure and its potential role in the bivalency of the toxin, we generated two mutants. The tyrosine within ICK1 was mutated to alanine (Y23 A), while the tyrosine in the linker was mutated to glycine (Y35G), the latter to maximise flexibility in this region. For these experiments, we used the WT peptide sequence to eliminate any effects of the ligation scar (retaining the additional N‐terminal glycine to aid cleavage of the fusion protein by TEV protease). This was feasible as the more costly ^13^C glucose was not required in these experiments and a SUMO‐fusion of the peptide, based on the reported MBP‐fusion, provided sufficient yields.^[24]^ First, the assignments of iDkTx were transferred to the WT DkTx sequence using a 3D ^15^N‐HSQC‐TOCSY experiment. The assignments were then transferred to each of the mutants (Y23A and Y35G) also using 3D ^15^N‐HSQC‐TOCSY experiments (Figure S4, Table S2).

Next ^15^N spin relaxation studies were carried out for each of the three peptides; WT, Y23A and Y35G versions of DkTx. These experiments are sensitive to motions on multiple timescales and can provide insights into changes in motion as a result of the introduced mutations that disrupt the aromatic stacking interaction. T_1_, T_2_ and heteronuclear NOE experiments were performed to extract parameters (Figure S5) used to model the dynamics of DkTx (Figure S5–S6 and Table S3). A T_1rho_ experiment (1.9 kHz spin‐lock field) was also performed to reveal the presence of μs‐ms timescale motion (Figure S5).

The spin relaxation data were used to perform model‐free analysis in order to relate the experimental data to protein dynamics. In all three peptides, we observed low heteronuclear NOE ratios (<0.5) around the C‐terminal end of the linker (residue 39–43) consistent with the random coil order parameters observed for this region in iDkTx and further indicating that the two lobes of the protein cannot be modelled by a single set of diffusion tensors. We therefore modelled each lobe of the peptide (on either side of residue 39) with an independent set of tensors (Table S3). All of the data were best fitted using an axially symmetric tensor model. In all three molecules, we find that ICK2 has a faster correlation time than ICK1. We also note that the correlation times of both ICK1 and ICK2 in the two mutants are consistently lower than what is observed in the WT for the corresponding ICK, indicating that the mutations reduce the effective size of the molecule supporting increased uncoupling of the two domains.

The model‐free analysis shows that for nearly all of the residues in DkTx (WT), complex models of motion are required to fit the experimental data, including contributions from either or both of fast internal motion (τ_e_) and chemical exchange (R_ex_). In general, we observe that ICK2 residues have lower R_2_ values suggesting a more compact structure than ICK1 (relaxation data provided in Supporting Data 1). This difference can be explained by the observation of increased chemical exchange in ICK1 residues both in the T_1rho_ data and the model‐free analysis (R_ex_), indicating that ICK1 is overall more dynamic than ICK2.

The presence of complex modes of motion is not surprising considering that in the ICK fold, the disulfide bonds (which account for >10 % of the amino acid content) are the only residues that are conserved.^[25]^ It is, therefore, common to find some conformational heterogeneity in the structure of these toxins, either due to conformational exchange in the (five) dihedral angles connecting the sidechains of cysteines^[26]^ or due to steric clashes of bulky sidechains, brought in proximity as a consequence of the covalent bonds stabilising the ICK pseudo knot.^[27]^ Here we, therefore, focus our analysis on the relative changes in dynamics in the regions of the proposed stabilising tyrosine stacking interaction, in the context of the introduced mutations.

Focusing on linker dynamics, we can compare the average order parameter in the three linker regions that retain residual order in the WT peptide (K33‐K36) and we find a clear drop in the order parameter from 0.97 in the WT peptide to 0.87 for both the Y23A and Y35G mutants. Specifically looking at the Y35 position we see that the order parameter of this residue drops from ≈1.0 to 0.72 in the Y23A mutant (G35 is 0.85). From this, we can conclude that the tyrosine stacking interaction stabilises the N‐terminal region of the linker, and disruption of this linker‐ICK1 interaction leads to increased linker disorder. We also note that the disruption of the tyrosine stack requires more complex modes of motion in the modelling, further supporting the stabilising role of the tyrosine stack on linker dynamics.

**Figure 3:**
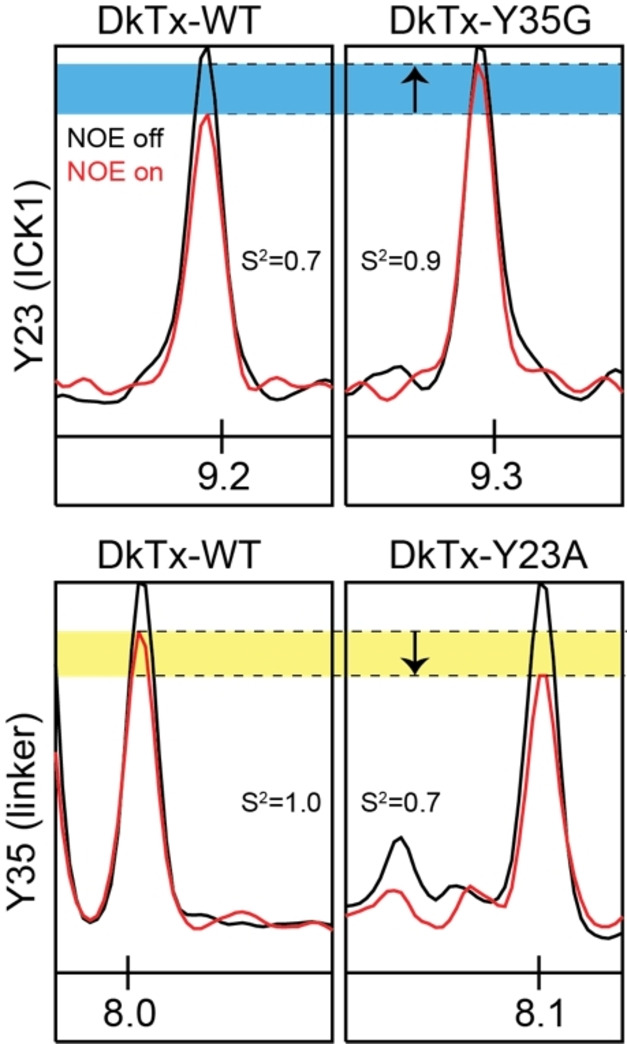
^15^N spin relaxation studies reveal increased disorder in the linker region when the tyrosine stacking interaction is disrupted. The changes in motion occur on the ns timescale and are apparent in the analysis of the heteronuclear NOE data that are sensitive to motions on this timescale. The Figure shows 1D traces from the 2D ^1^H‐^15^N correlation spectrum in the presence or absence of the heteronuclear NOE. Mutation of the linker‐tyrosine results in increased order in ICK1 (Y23—top), while mutation of the ICK1‐tyrosine results in increased disorder in the linker (Y35—bottom).

In the Y23 region of ICK1, the changes in motion due to the introduced mutations are reversed, with the mutations causing an increase in order (S^2^ of residue 23 going from 0.69 to 0.77 and 0.95, comparing WT to Y23A and Y35G mutants respectively). This suggests that the tyrosine stacking interaction introduces local disorder in ICK1 which is relaxed into a more ordered state when the stack is disrupted. This is evidence of an interesting case of entropy‐entropy compensation, where the enhanced disorder observed in the linker as a consequence of the introduced mutations leads to the stabilisation of the affected region in ICK1 (Figure 3).

Finally, we can look for evidence of changes in conformational exchange in the μs‐ms timescale by comparing the T_2_ and T_1rho_ values. The former is sensitive to faster (ns) scale motion and the latter to slower exchange processes (μs‐ms). For the WT peptide we find that there is indeed a notable exchange component in the ns timescale at residue Y23, C32 and K33—high T_2_ values compared to T_1rho_ values. There is, however, no clear evidence of (μs‐ms) timescale motion in the regions involved in the tyrosine stacking (there are some elevated T_1rho_ values near the second cysteine of the peptide). Comparing the relaxation parameters between the WT and mutant DkTx peptides, the differences are most apparent in the ns timescale, and follow the trends described above from the model free analysis. There is some evidence of decreased (μs‐ms) timescale motion in the mutants near residue 36 but these changes are modest in comparison to those observed on the faster timescale. From this we conclude that the changes in dynamics, in the region of interest, due to the introduced mutations, occur on the faster ns timescale.

Taken together, the relaxation data suggest that disruption of the tyrosine stacking interaction leads to increased linker disorder due to motion in the ns timescale, which also results in increased independent rotational diffusion of the individual domains. The enhanced dynamics in the linker further lead to reduced dynamics in ICK1, also on the ns timescale. We interpret this as stemming from the loss of a constraint (aromatic stacking) in this region of the peptide, which is already constrained by a disulfide‐bond and the β‐sheet core of the ICK fold.

### Domain‐linker interaction enhances bivalency

**Figure 4:**
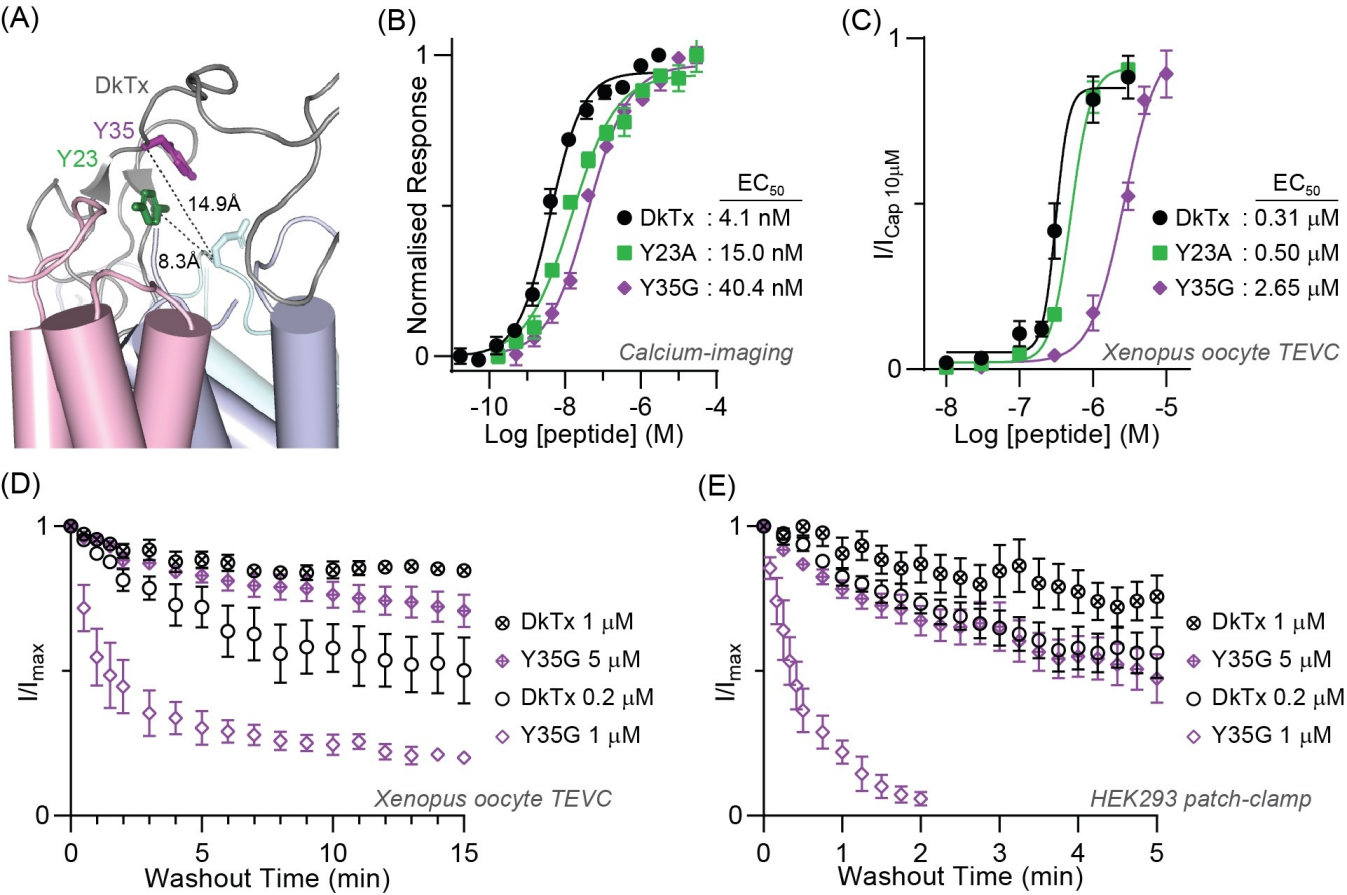
Functional data validates the proposed simultaneous ICK1 and ICK2 binding model of DkTx to TRPV1 that provides high avidity. (A) Close‐up view of the cryo‐EM structure with DkTx (grey) bound to TRPV1 (PDB: 7L2U) showing the mutated tyrosine residues likely do not make direct channel contacts. Side chain sticks highlight the DkTx residues that form the tyrosine stack, and their distance to the closest TRPV1 residue (E651) is measured. (B) Tyrosine mutants (Y23A and Y35G) have reduced activity compared to DkTx as determined by calcium‐imaging with HEK293 cells stably expressing hTRPV1. Values are normalised to the maximal response evoked for each peptide. Hill equation fits to the data give the EC_50_ in panels B and C. (C) Concentration‐response analyses of two‐electrode voltage‐clamped (TEVC) *Xenopus* oocytes, expressing rat TRPV1 similarly show a loss in activity for tyrosine mutants relative to DkTx. Oocytes were held at −60 mV and data normalised to currents elicited by 10 μM capsaicin. (D) Reversibility of DkTx and Y35G evoked rat TRPV1 response at the indicated peptide concentrations using TEVC oocytes. Data points represent the fraction of currents remaining during the washout as a ratio to the maximal current evoked by each sample. (E) Washout shown as fractional current determined by whole‐cell patch clamp recordings from HEK293 cells expressing TRPV1 (voltage‐clamped at −80 mV). Note the increased reversibility of Y35G at the lower sub‐EC_50_ concentration that is different from the persistent currents from DkTx at a sub‐EC_50_ concentration. See Figure S7 for the activity of peptides in patch‐clamp experiments relative to capsaicin. The time constant for decay was not fitted and calculated in panels D and E, as it could not be accurately determined for data sets with minimal washout over the experimental time course. All data are mean ± standard error of the mean (*n*=5–7).

Analysis of the structure and dynamics of DkTx, reveals the pre‐organisation of the quaternary structure of this toxin, involving a stabilising interaction between the linker and the first domain of the toxin. The reported structures of DkTx bound to the TRPV1 channel under different conditions, reveal that the tyrosine stacking interaction, while present in the bound state does not contribute to channel binding (Figure 4A). This provides an opportunity to relate any changes in mutant activity to changes in toxin structure or dynamics rather than direct disruption of interactions with the channel.

Previous work has shown that the individual domains of the toxin are less potent than the full‐length toxin (5‐ and 50‐fold reduced potency of ICK2 and ICK1 respectively).^[2]^ Furthermore, it has been shown that mutations that lengthen the linker using a dynamic linker (poly‐Gly) also lead to reduced potency (15‐fold reduced activity).^[10]^ It has been proposed that DkTx binding is a sequential event of ICK2 and then ICK1 binding, however, given that DkTx is more potent than either ICK1 or ICK2 alone, it is more likely that the enhanced potency is the product of the binding affinities (in the picomolar range) modulated by the available population in this state. The modest enhancement (5–50 fold) suggests that this state is, even in WT‐DkTx, sparsely populated, and further disruption of any domain‐preorganisation will further reduce the available “bivalent” population such that it may have an apparent affinity below that of the most potent single domain (ICK2).

Provided that disruption of the tyrosine stacking interaction causes a loss of domain coupling and reduced bivalent engagement, we would expect a concomitant loss of binding in the order of 5‐ to 50‐fold. Indeed, we find that Y23 A and Y35G mutants have reduced potency of 3.7‐ and 10‐fold with calcium‐imaging experiments (Figure 4B), and 1.7‐ and 8.5‐fold in two‐electrode voltage‐clamped (TEVC) *Xenopus* oocytes (Figure 4C), supporting that the tyrosine stacking interaction is important for bivalent channel engagement. We further examined the avidity of the less potent Y35G mutant by studying the reversibility of channel activation compared to DkTx in oocytes and patch‐clamped HEK293 cells. Currents elicited by saturating concentrations of DkTx (1 μM) or Y35G (5 μM) resulted in persistent activation with minimal washout over several minutes, indicative of a bivalent binding conformation (Figure 4 D and E; Figure S7). As previously shown,^[2]^ this activity is maintained with sub‐saturating concentrations of DkTx (0.2 μM), with >50 % of current remaining after extended washout. In contrast, sub‐saturating Y35G (1 μM) evoked currents showed substantial washout within 3 minutes. We conclude that once the bivalent peptide is bound, there is only a small reduction in dissociation kinetics, suggesting that linker pre‐organisation has a less significant impact on dissociation compared to channel engagement.

The functional data supports a model where domain‐linker interactions stabilise a bivalent‐binding‐competent DkTx conformation. In a simultaneous (ICK1+ICK2) bivalent binding model, the encounter complexes leading to two‐domain binding must contain a certain population of preconfigured DkTx ICK1 and ICK2 orientations to yield irreversible binding. Depopulating this preconfigured state through disruption of linker order would increase the apparent EC_50_ of irreversible binding. If this EC_50_ exceeds that of the more potent single domain binding of ICK2 only (≈1 μM),^[2]^ it would result in the data we see for Y35G. Sequential binding may also explain this by linker preorganisation populating binding competent ICK1 orientations following ICK2 binding, but it is then unclear why DkTx should have a lower EC_50_ than ICK2 alone. Our simultaneous binding model better interprets the available data and differs from the previously proposed sequential binding model, providing new insights into the mechanism of bivalency in this emerging class of multi‐domain toxins.

The data further highlights some important principles of multidomain binding when applied to peptide engineering. In cases where two functionally active peptides with distinct receptor binding sites are conjugated using an unstructured linker—such as PEG or poly‐glycine—it is unlikely that this will result in a dimer with enhanced potency (compared to the constituent domains). High‐avidity binding may still be observed but this is likely to occur when the dimer is applied at a concentration higher than that needed to elicit a response from the more potent constituent domain (at or above saturating concentrations). Instead, if the aim is to produce an engineered peptide that is more potent and avid than either constituent domain, these must be linked by a linker that pre‐configures them to populate an orientation where bivalent engagement can be achieved. Such engineering requires detailed knowledge of the binding of each domain to the receptor and must be combined with a library of linkers that stabilise different distances and orientations. A suitable source of the latter may be found in the curated linker regions of naturally occurring multidomain peptides contained within the SCREPyard database.^[5]^ Combination of such a template library with computational modelling would provide an attractive approach to engineering bivalent peptides.

## Conclusion

The discovery of multidomain toxins with a bivalent mode of action suggests that these confer an evolutionary advantage in predation or defence compared to their single domain counterparts. In cases such as DkTx, where the two domains bind to adjacent non‐overlapping receptor sites, we find that structural elements in the linker serves to reduce linker dynamics and pre‐organise the two domains in conformations biased for simultaneous receptor engagement. This mechanism enhances the potency of the peptide and reduces the concentration at which avid receptor binding occurs. In contrast, we find that domain preorganisation is less important for the reversibility of the persistent response after the onset of bivalent engagement.

Our analysis of the structure and dynamics of DkTx, further reveals evidence of disorder in the region connecting the linker to the second domain. This may reflect ongoing evolution in the toxin, where further stabilisation of the linker may yield more potent bivalent toxins that display receptor avidity at much lower concentrations (up to a theoretical EC_50_‐limit in the pM range). Finally, our findings provide insights into how multidomain peptides and proteins may be engineered for enhanced potency and avidity through introduction of stabilising elements such as the linker‐domain interactions described in this study.

## Conflict of interest

The authors declare no conflict of interest.

1

## Supporting information

As a service to our authors and readers, this journal provides supporting information supplied by the authors. Such materials are peer reviewed and may be re‐organized for online delivery, but are not copy‐edited or typeset. Technical support issues arising from supporting information (other than missing files) should be addressed to the authors.

Supporting Information

## Data Availability

The data that support the findings of this study are available from the corresponding author upon reasonable request.
