# Supplementary material for: Structural Basis of the Bivalency of the TRPV1 Agonist DkTx
Source: Angew Chem Int Ed Engl. 2023 Dec 12;63(3):e202314621. doi: 10.1002/anie.202314621 (PMC10952689; doi:10.1002/anie.202314621)
Supplement: Supplementary file 1 — Supporting Information [file ANIE-63-0-s001.pdf]

Angewandte Chemie
Eine Zeitschrift der Gesellschaft Deutscher Chemiker

Supporting Information

Structural Basis of the Bivalency of the TRPV1 Agonist DkTx

*V. Ramanujam, T. Crawford, B. Cristofori-Armstrong, J. R. Deuis, X. Jia, M. J. Maxwell, S. Jami, L. Ma, I. Vetter, M. Mobli**

Table of Contents

1. Supporting Experimental Procedures 3

2. Supporting Figures 10

3. Supporting Tables 17

4. Supporting References 22

1. Supporting Experimental Procedures

Mutation of catalytic cysteine in the C-extein sequences

The plasmids pJJDuet30 and pSFBAD09 containing the respective N- and C- terminal DNA polymerase III split inteins from *Synechocystis sp.* PCC6803, and pSKDuet01 and pSKBAD2 containing the split inteins from *Nostoc punctiforme* were obtained as a gift from Prof. Hideo Iwai (Addgene plasmids 11962, 11963, 12172, 15335 respectively).[1] The first residue of the native C-extein sequences (pSFBAD09 and pSKBAD2) were mutated using the Q5 Site-Directed Mutagenesis (New England Biolabs) protocol with synthetic oligonucleotides encoding serine and threonine. All DNA sequences were confirmed by Sanger DNA sequencing, carried out at the Australian Genome Research Facility (AGRF, Brisbane).

In vivo trans-splicing and purification of modified intein systems

50 ml of Lysogeny broth (LB) supplemented with 100 µg/ml ampicillin and 50 µg/ml kanamycin was inoculated with BL21(*DE3*) cells harboring combinations of split inteins (i) pJJDuet30/pSFBAD09, (ii) pJJDuet30/pSKBAD2, (iii) pSKDuet01/pSKBAD2, and (iv) pSKDuet01/pSFBAD09. The cultures were grown at 37°C to a cell density of OD_{600} 0.5 and induced with 0.04% L-arabinose for the expression of the C-terminal split intein. After 35 min, the N-terminal split intein was induced with 0.5 mM of IPTG. After 3 hours the cells were harvested by centrifugation at 4000 x g for 15 min. The harvested cells were resuspended in 1 ml of buffer A (30 mM Tris, 300 mM NaCl pH 8.0) and lysed using CelLytic™ B Cell Lysis Reagent (Sigma Aldrich). The cell debris was removed from the protein solution by centrifugation for 15 min at 14,000 x g. The supernatant was loaded onto a Ni-NTA resin (Cytiva) spin column equilibrated with buffer A and centrifuged for 1 min at 4000 x g. The column was washed twice with 700 µl of buffer B (30 mM Tris, 300 mM NaCl, 30 mM imidazole, pH 8.0). The bound protein was eluted from the spin column using 200 µl of buffer C (30 mM Tris, 300 mM NaCl, 250 mM imidazole, pH 8.0).

Linker design for intein DkTx (iDkTx) construct

The native (WT) DkTx linker has the following sequence: KKYKPYVPVTTN. The initial construct had a linker included a mixture of intein linker residues and DkTx residues (KKYKGSSFNGTN). This His-tagged protein was used to optimise the expression and purification protocols (Figure S1). The final iDkTx construct had a linker sequence of KKYKPYVPVTTSFN, where an SF was inserted in the position where previously a HYR sequence (KKYKPYVPVTTHYRN) had been inserted and shown to not affect activity.[3] In this construct we also introduced a TEV protease cleavage site to remove the N-terminal His-tag, yielding a construct that started with a non-native G. The insertion of an SF (and N-terminal glycine) did not interfere with the activity or bivalency of the peptide.

Cloning of DkTx domains into intein vectors

A gene corresponding to the ICK1 region (domain 1) of DkTx was cloned into the pSKDuet01 using *NcoI* and *BamHI* restriction sites to yield pSspN-ICK1. The ICK2 region (domain 2) of DkTx was cloned into the pSFBAD09 plasmid using *NdeI* and *HindIII* restriction sites to yield pSspC-ICK2 with serine as the first extein residue.

Expression of in vivo ligated iDkTx

SHuffle® T7 Express Competent E. coli[2] (New England Biolabs) cells were co-transformed with the plasmids pNpuN-ICK1 and pSspC-ICK2. The transformed cells were inoculated in LB media supplemented with 100 µg/ml

ampicillin and 50 µg/ml kanamycin and grown overnight at 30°C. Once the OD_{600} reached 0.5, the culture was induced with 0.04% L-arabinose for 30 min followed by addition of 0.5 mM IPTG and incubation for 18 h at 20°C. The cells were harvested and frozen at -80°C.

Expression of segmentally labeled iDkTx

The pNpuN-ICK1/pSspC-ICK2 co-transformed SHuffle cells were grown in 1 litre of LB medium containing 100 µg/ml ampicillin and 50 µg/ml kanamycin at 30°C. Once the OD reached 0.4, the cells were spun down for 20 min at 4000 x g and resuspended in 250 ml of M9 medium containing NH_4Cl (1 g/L) and glucose (4 g/L) as the sole nitrogen and carbon sources, supplemented with 100 µg/ml ampicillin and 50 µg/ml kanamycin. The cells were then induced with 0.04% L-arabinose for 3 h. Then, the cells were spun down for 20 min at 4,000 x g and washed with 250 ml of M9 medium to remove the residual L-arabinose, the washed cells were resuspended in 250 ml of M9 medium with $^{15}NH_4Cl$ (1 g/L) and glucose (4 g/L) as the sole nitrogen and carbon sources, supplemented with 100 µg/ml ampicillin and 50 µg/ml kanamycin. The final resuspended culture in M9 media was induced with 0.5 mM IPTG and incubated for 18 h at 20°C. The cells were harvested and frozen at -80°C.

Expression of uniformly isotope labeled iDkTx

The pNpuN-ICK1/pSspC-ICK2 co-transformed SHuffle cells were grown in 1 litre of LB medium containing 100 µg/ml ampicillin and 50 µg/ml kanamycin at 30°C. Once the OD_{600} reached 0.4, the cells were spun down for 20 min at 4000 x g and resuspended in 250 ml of M9 minimum medium with $^{15}NH_4Cl$ (1 g/L) and ^{13}C-glucose (4 g/L) supplemented with 100 µg/ml ampicillin and 50 µg/ml kanamycin. The cells were then induced with 0.04% L-arabinose for 30 min followed by addition of 0.5 mM IPTG and further grown for 18 h at 18°C. The bacterial cells were harvested by centrifugation at 4000 x g for 30 min at 4°C.

Purification of in vivo ligated iDkTx

The harvested cells were resuspended in lysis buffer (30 mM Tris, 300 mM NaCl, 10 mM imidazole pH 8.0), lysed by sonication and centrifuged for 40 min at 40000 x g. The supernatant was filtered through 0.45 µm filter and loaded onto a 5 ml HisTrap FF (Cytiva) column equilibrated with lysis buffer. The column was extensively washed with wash buffer (30 mM Tris, 300 mM NaCl, 40 mM imidazole pH 8.0) and bound proteins eluted with elution buffer (30 mM Tris, 300 mM NaCl, 250 mM imidazole pH 8.0). The eluted proteins were buffer-exchanged with Tris-buffer (400 mM Tris, 1 mM EDTA, 0.5% Triton X-100, 2.5 mM GSH and 0.25 mM GSSG) and incubated overnight. The protein mixture containing the *in vivo* ligated DkTx and other intein products were acidified with 0.05% trifluoroaceticacid (TFA) and filtered through a 0.45 µm filter. The sample was then applied to a Zorbax C3-semi prep column (Agilent) using reverse-phased HPLC running at 3 ml/min with a linear gradient of 5 – 80% acetonitrile (0.043% TFA) in water (0.05% TFA) over 70 min while monitoring elution by UV absorption at 214 nm and 280 nm. The fractions containing iDkTx were collected and lyophilised. iDkTx was separated from the $(His)_6$ tag by Tobacco Etch Virus (TEV) protease. The cleavage was performed by adding TEV protease (1 mg/ml) to the protein solution (at an A280 ratio of 1:10) in a redox buffer (30 mM Tris, 2.5 mM GSH, 0.25 mM GSSG pH 8.0) and incubated at 25°C overnight. The iDkTx was separated from the cleaved mixture by RP-HPLC using a Zorbax C3-semi prep column (Agilent). Fractions containing iDkTx were pooled, lyophilised and stored at -20°C.

Cloning of native DkTx and tyrosine mutants into a SUMO-fusion vector

The MBP gene in the plasmid pLIC-MBP-DkTx[4] was replaced by a SUMO gene with *Nde*I/*Kpn*I restriction enzyme digestion to generate SUMO-DkTx. The SUMO gene flanked by *Nde*I and *Kpn*I recognition sites was obtained by a PCR amplification. The coding gene for DkTx-Y23A and DkTx-Y35G was generated by the PCR-based mutagenesis in the template plasmid of SUMO-DkTx.

Expression of full-length native DkTx

The SUMO-DkTx constructs (WT, Y23A, Y35G) were expressed in SHuffle® T7 Express Competent E. coli (New England Biolabs). The freshly transformed colonies were used to inoculate 15 ml of LB media supplemented with 100 µg/ml ampicillin and grown overnight at 25°C with shaking (200 rpm). The overnight culture was added to 1 L of LB media supplemented with 100 µg/ml ampicillin and grown 30°C with shaking (250 rpm). Once the cell density reached OD_{600} 0.8-1.0, the cells were induced with 0.2 mM IPTG. Induction was performed for 5 hours at 30°C. The cells were then spun down (8000 x g, 15 min), flash frozen in liquid nitrogen, and stored at -80°C.

Expression of ^{15}N labelled full-length native DkTx

SHuffle® T7 Express Competent E. coli containing SUMO-DkTx constructs (WT, Y23A, Y35G) were grown in 2 L of LB media supplemented with 100 µg/ml ampicillin as previous described. Once the cell density reached OD_{600} 0.6-0.8, the cells were pelleted by centrifugation (4000 x g, 10 min). The cell pellet was resuspended in 100 ml M9 salts (110 mM KH_2PO_4, 450 mM Na_2HPO_4, 85 mM NaCl) and pelleted by centrifugation (4000 x g, 10 min). The cells were then resuspended in 1 L of M9 minimal media containing M9 salts, 1X BME vitamins solution (Merck), 2 mM $MgSO_4$, 0.1 mM $CaCl_2$, 1% (w/v) glucose, 18.7 mM $^{15}NH_4Cl$, 100 µg/ml ampicillin. The cells were cultured for 30 minutes at 30°C before inducing with 0.2 mM IPTG. After 5 hours induction the cells were harvested by centrifugation (8000 x g, 15 min), flash frozen in liquid nitrogen, and stored at -80°C.

Purification of full-length native DkTx

The over-expressed cell pellet from 1 L of culture was resuspended in 40 ml binding buffer (20 mM Tris, 150 mM NaCl pH 7.8). The cells were lysed by ultrasonication, using a VCX 750 Sonicator and 13 mm probe set to 40% amplitude (5 sec on/10 sec off), 20 min total time, on ice inside a 4°C refrigerator. The lysed solution was centrifuged (40000 x g, 30 min) and the supernatant applied to a 5 ml HisTrap FF column (Cytiva), pre-equilibrated with binding buffer. The column was washed with 5 CVs of wash buffer (20 mM Tris pH 7.8, 150 mM NaCl, 20 mM imidazole) and eluted with 5 CVs of elution buffer (20 mM Tris pH 7.8, 150 mM NaCl, 250 mM imidazole). The eluted protein was buffer exchanged to binding buffer using a 10 kDa MWCO Amicon Ultra centrifugal filter (Merck) and concentrated to 10 ml. 1 mg of TEV protease and 10 mM DTT was added to the solution and incubated at 37°C for 3-5 hrs with shaking (100 rpm). The cleaved sample was centrifuged (8000 x g, 10 minutes) and the supernatant discarded. The precipitation was dissolved in 10 ml of 6 M guanidine hydrochloride in binding buffer containing 10 mM DTT and incubated at 37°C overnight. The dissolved precipitate solution was acidified with TFA (final concentration 1%) and centrifuged (8000 x g, 15 minutes). The supernatant was filtered using a 0.45 µm syringe filter and applied to a Zorbax C3-semi prep reverse-phased HPLC column (Agilent) at a flow rate of 3 ml/min. The protein was eluted with a linear gradient of 5 – 80% acetonitrile (0.043% TFA) in water (0.05% TFA) over 60 min, monitoring UV absorbance at 214 nm and 280 nm. The fractions containing DkTx, determined by MALDI-TOF

mass spectrometry, were collected and lyophilised. The lyophilised DkTx was dissolved in 1 ml of 50% acetonitrile in water, diluted to 10 µM with refolding buffer (400 mM Tris pH 8.0, 1 mM EDTA, 2.5 mM GSH, 0.25 mM GSSG, 0.1% (v/v) Triton X-100), and incubated at 4°C for 48 hrs as previously described.[5] The refolded DkTx solution was acidified with TFA (final concentration 1%), and applied to a Zorbax C3-semi prep reverse-phase HPLC column (Agilent). The fractions containing DkTx were collected and lyophilised. The refolded DkTx fractions were dissolved in 20 mM phosphate buffer (pH 5.8) containing 5% D_2O. ^1H spectra were recorded for each refolded DkTx fraction on a Bruker (Avance III) spectrometer operating at nominal ^1H frequency of 700 MHz equipped with a cryoprobe. Fractions that displayed peak dispersion in the amide region were re-purified by reverse-phase HPLC and lyophilized and stored at -20°C for later use in electrophysiology experiments.

NMR measurements

The NMR sample of DkTx contains [U-^{13}C,^{15}N] DkTx-construct4 in 20 mM phosphate buffer (pH 5.8) with 0.02% NaN_3 and 10 µM 2,2-Dimethyl-2-silapentane-5-sulfonate sodium salt (DSS, Sigma-Aldrich) in a 95/5% (v/v) H_2O/D_2O mixture. All of the following spectra were recorded with a 300 µM sample concentration at 25°C in a volume of 300 µl (5 mm NMR cell, susceptibility matched to water – Shigemi) on a Bruker spectrometer operating at 900.1 MHz ^1H resonance frequency equipped with triple resonance cryoprobe with an interscan delay of 1.0 s and a NOE mixing time of 200 ms where applicable. The non-uniform sampling schedules were designed to follow the decay profile of the indirect dimensions. Where decay rates equivalent to a line width of 15 Hz were used for non-constant time (CT) dimensions and no decay was used for CT dimensions. All sampling schedules were generated using the Sched3d software.[6]

A high-resolution 2D [^{15}N-^1H] HSQC spectrum was recorded for assigning the backbone amide resonances. The time domain matrix consisted of 256 (t1, ^{15}N) ´ 2048 (t2, ^1H) complex points with acquisition times of 50.1 ms (t1) and 75.8 ms (t2) using 8 scans per FID with the ^{15}N carrier at 117.0 ppm.

A 3D HNCO[7] spectrum was recorded for assigning the carbonyl (^{13}C') resonances. The time domain matrix consisted of 100 (t1, ^{13}C) ´ 100 (t2, ^{15}N) ´ 2048 (t3, ^1H) complex points with acquisition times of 22.1 ms (t1), 19.6 ms (t2) and 81.1 ms (t3) using 16 scans per FID with the ^{13}C and ^{15}N carriers at 175.0 and 117.0 ppm respectively. The spectrum was acquired using non-uniform sampling (NUS) with 140 data coordinates.

A 3D CBCA(CO)NH[8] spectrum was recorded for assigning the $^{13}C_\alpha$ and $^{13}C_\beta$ resonances. The time domain matrix consisted of 128 (t1, ^{13}C) ´ 100 (t2, ^{15}N) ´ 2048 (t3, ^1H) complex points with acquisition times of 3.5 ms (t1), 19.6 ms (t2) and 81.1 ms (t3) using 56 scans per FID with the ^{13}C and ^{15}N carriers at 48.0 and 117.0 ppm respectively. The spectrum was acquired using NUS with 400 data coordinates.

A 3D HNCACB[9] spectrum was recorded for assigning the intra and inter-residue $^{13}C_\alpha$ and $^{13}C_\beta$ resonances together with the 3D CBCA(CO)NH experiment. The time domain matrix consisted of 128 (t1, ^{13}C) ´ 100 (t2, ^{15}N) ´ 2048 (t3, ^1H) complex points with acquisition times of 3.5 ms (t1), 19.6 ms (t2) and 81.1 ms (t3) using 80 scans per FID with the ^{13}C and ^{15}N carriers at 48.0 and 117.0 ppm respectively. The spectrum was acquired using NUS with 650 data coordinates.

A 3D HBHA(CO)NH[8] spectrum was recorded for assigning the $^1H_\alpha$ and $^1H_\beta$ resonances. The time domain matrix consisted of 128 (t1, ^1H) ´ 100 (t2, ^{15}N) ´ 2048 (t3, ^1H) complex points with acquisition times of 7.9 ms (t1), 19.6 ms (t2) and 71.0 ms (t3) using 40 scans per FID with the ^{15}N carrier at 117.0 ppm. The spectrum was acquired using NUS with 600 data coordinates.

A 3D HCCH-TOCSY[10] spectrum was recorded for assignment of all aliphatic ^{1}H and ^{13}C resonances. The time domain matrix consisted of 128 (t1, ^{13}C) ´ 100 (t2, ^{15}N) ´ 2048 (t3, ^{1}H) complex points with acquisition times of 7.4 ms (t1), 2.8 ms (t2) and 81.1 ms (t3) using 16 scans per FID with the ^{13}C and carrier at 42.0 ppm. The spectrum was acquired using NUS with 1450 data coordinates.

A 3D ^{13}C edited NOESY[11] spectrum was recorded for assigning ^{1}H-^{1}H NOEs from aliphatic CH group to nearby hydrogens. The time domain matrix consisted of 110 (t1, ^{1}H) ´ 82 (t2, ^{13}C) ´ 2048 (t3, ^{1}H) complex points with acquisition times of 5.1 (t1), 2.5 (t2) and 75.8 (t3) ms using 16 scans per FID with the ^{13}C carrier at 42.0 ppm.

A 3D ^{13}C edited NOESY[11] spectrum was recorded for assigning aromatic residues and the correpoding^{1}H-^{1}H NOEs. The time domain matrix consisted of 92 (t1, ^{1}H) ´ 44 (t2, ^{13}C) ´ 2048 (t3, ^{1}H) complex points with acquisition times of 4.3 (t1), 3.7 (t2) and 75.8 (t3) ms using 16 scans per FID with the ^{13}C carrier at 126.0 ppm.

A 2D high-resolution [^{13}C-^{1}H] HSQC spectrum was recorded. The time domain matrix consisted of 256 (t1, ^{13}C) ´ 2048 (t2, ^{1}H) complex points with acquisition times of 7.1 (t1) and 75.8 (t2) ms using 16 scans per FID with the ^{13}C carrier at 42.0 ppm.

A 3D ^{15}N edited NOESY[12] spectrum was recorded for assigning NOEs from one NH group to nearby hydrogens. The time domain matrix consisted of 110 (t1, ^{1}H) ´ 72 (t2, ^{15}N) ´ 2048 (t3, ^{1}H) complex points with acquisition times of 5.1 (t1), 14.1 (t2) and 81.1 (t3) ms using 16 scans per FID with the ^{15}N carrier at 117.0 ppm.

The non-uniformly sampled spectra were reconstructed using the automated maximum entropy method.[13] All the spectra were processed using the Rowland NMR toolkit[14] and analysed using CCPNMR[15] program.

Spin Relaxation Data Analysis

NMR experiments for measuring longitudinal (R_1), transverse relaxation rates (R_2) and ^{1}H-^{15}N steady-state NOE were acquired using interleaved pulse sequence on a 900 MHz Bruker spectrometer equipped with z-gradient cryoprobe. Data were acquired at 298K using a 200 µM sample of ^{15}N labeled DkTx. R_1 spectra were recorded with 8 delay times (10, 20, 60*, 100, 200, 400, 600* and 1200 ms), while R_2 spectra were recorded with 8 delay times (16, 31*, 63, 125, 157, 188*, 220, 251 ms). The $R_{1\rho}$ spectra were collected using a spin lock RF field strength of 1.9 kHz and relaxation delays of 0, 4, 12, 24*, 48, 72, 96*, 120 and 144 ms. In the above-mentioned experiments, delays marked with an asterisk were recorded twice to check the reproducibility of the data. ^{15}N-{^{1}H} NOE experiment was collected in an interleaved fashion, alternating between free induction decays with and without 5 s of proton saturation. The spectra were processed using NMRPipe [1] and were analysed using Sparky [2]. The R1, R2 and $R_{1\rho}$ relaxation data were fit using single exponential functions in Sparky. The effective R_2 values were calculated from R_1 and $R_{1\rho}$ using the relation [3].

$$R_2 = R_{1\rho} / \sin^2\theta - R_1 / \tan^2\theta$$

with $\tan\theta = \omega_1/\Omega$, where ω_1 is the spin-lock RF field strength and Ω the offset from the ^{15}N carrier.

The heteronuclear ^{15}N-{1H} NOE values were obtained by taking the ratio of two experiments recorded with and without ^{1}H saturation.

The order parameter (S^2), the effective correlation time for fast internal motions (τ_e), and the rotational correlation time (τ_m) were obtained by fitting the R1, R2, and NOE values within the framework of the Lipari-Szabo formalism [4]. The R_2/R_1 ratio for residues, whose relaxation is not affected by fast internal motions on the picosecond time scale, is approximately independent of the internal motion and provides an initial estimation for the reorientation

time of each NH vector with global tumbling, i.e., the τ_m of the peptide. For this purpose, residues with the R_2/R_1 ratio falling outside one standard deviation of the mean, as well as residues with NOE < 0.65, were disregarded. The selection of the most appropriate diffusion tensor model was performed using the programs quadric diffusion [5] and R2R1 diffusion [6], assuming isotropic, axially symmetric, and fully anisotropic molecular diffusion models. All the models were applied to the individual domains of DkTx (ICK1 and ICK2) using the data comprising 8 (or 9 for WT) and 12 (or 11 for Y35G) T_1/T_2-values for all the variants of ICK1 and ICK2, respectively. The parameters obtained by fitting these models were summarised in Table S3. Subsequently, the dynamics parameters were calculated for all the residues using the Lipari-Szabo model-free approach and the axially symmetric anisotropic diffusion model using the FASTModelfree software package [7].

Calcium fluorescence assay

HEK293 cells stably expressing tetracycline-inducible hTRPV1 channel (TRPV1-HEK293 cells, a kind gift from Prof. Peter McIntyre, RMIT University, Australia) were cultured in Dulbecco's Modified Eagle Medium (DMEM) medium supplemented with 10% FBS, 2 mM glutamine, 1% (v/v) non-essential amino acids, 5 μg/ml of blasticidin and 100 μg/ml of hygromycin, at 37°C with 5% CO_2. To induce the expression of TRPV1, 1 μg/mL tetracycline was added to the media 12–16 h before recording.

HEK293 cells stably expressing hTRPV1 were plated 24 h before the assay on 384-well black-walled imaging plates coated with poly d-lysine (ViewPlate-384, Perkin Elmer, Victoria, Australia) at a density of 10,000–30,000 cells/well and loaded with Calcium 4 no-wash dye (Molecular Devices) diluted in physiological salt solution (PSS; 140 mM NaCl, 11.5 mM glucose, 5.9 mM KCl, 1.4 mM $MgCl_2$, 1.2 mM NaH_2PO_4, 5 mM $NaHCO_3$, 1.8 mM $CaCl_2$, 10 mM HEPES) and incubated for 30 min at 37 °C. Peptide samples were diluted in PSS with 0.1% bovine serum albumin (BSA) at the concentrations stated and added using the $FLIPR^{TETRA}$. Changes in fluorescence were assessed using the $FLIPR^{TETRA}$ (excitation 470–495 nm, emission 515–575 nm) every 1 s for 300 s after the addition of peptides. Camera gain and intensity were adjusted for each plate of cells yielding a minimum of 1000 arbitrary fluorescence units (AFU) as a baseline fluorescence value. TRPV1 channel activity was stimulated by DkTx peptides for 400 s before 5 μM capsaicin was added as a loading control. Fluorescence responses were quantified by the maximum increase in fluorescence computed using ScreenWorks 3.2.0.14.

Automated patch-clamp

Patch-clamp recordings were performed with an automated whole-cell patch-clamp electrophysiology system (QPatch 16X; Sophion, Ballerup, Denmark) using the above described tet-inducible human TRPV1-expressing HEK293 cells. The extracellular solution comprised 140 mM NaCl, 4 mM KCl, 1 mM $MgCl_2$, 2 mM EGTA-Na and 10 mM HEPES-Na, pH 7.4, and the intracellular solution comprised 50 mM CsCl, 60 mM CsF, 10 mM NaCl, 20 mM EGTA-Na and 10 mM HEPES-Cs, pH 7.2. The membrane potential was held at 0 mV, and currents were elicited by a protocol consisting of a 300-ms step to +80 mV followed by a 200-ms step to −80 mV at 1 s intervals. The elicited currents were sampled at 25 kHz and filtered at 4 kHz. Experimental data were analysed using QPatch Assay Software version 5.0 (Sophion), Igor Pro (WaveMetrics) and Prism 8 (GraphPad).

Whole cell patch-clamp

Separate HEK293 cells stably expressing human TRPV1 were used for manual patch-clamp recordings. These cells were cultured in DMEM supplemented with 10% Fetal Bovine Serum (Gibco), and passaged every 2–3 days with Accutase (Merck) until pass #20. For electrophysiology, cells were plated onto coverslips coated with 0.1% Poly-L-lysine followed by 1:50 ECM gel (Merck) 24−48 before recording.

Whole cell patch-clamp recordings were performed at room temperature (~23°C) using a Multiclamp 700B amplifier and digitised with a Digidata 1550B and pClamp 10.7 software (Molecular Devices). Data were digitised at 10 kHz and filtered at 2 kHz. Recordings were corrected for pipette capacitance, whole-cell capacitance, and series resistance compensated to 70%. Patch pipettes were pulled from borosilicate glass to a final resistance of 2−6 MΩ when filled with (in mM) 150 NaCl, 10 $MgCl_2$, 10 EGTA, 10 HEPES. The bath solution contained (in mM) 150 NaCl, 10 EGTA, 10 HEPES. Solutions were 300 mOsm with mannitol and pH 7.4 with NaOH. Cells were held at −60 mV for all experiments. For voltage ramps, cells were stepped to −80 mV for 50 ms, then ramped to +80 mV over 1 s, held at +80 mV for 50 ms, and returned to the holding potential. The time between each ramp starting was 5 s. Solutions were applied to cells via a local gravity fed perfusion with internal diameter of 250 mm that was placed 50−100 mm from the patched cell.

Two-electrode voltage-clamp

Rat Trpv1 cloned into pcDNA3.1 plasmid was linearised with NotI restriction enzyme and used as template for cRNA synthesis using T7 polymerase (mMessage mMachine Kit; Life Technologies). Stage V/VI oocytes were injected with 100–250 ng total RNA per oocyte, then incubated at 17°C for 1–3 days in 50% Leibovitz's L-15 medium (Gibco), supplemented with 25 µg/ml gentamicin, 25 µg/ml streptomycin, and 2.5% foetal horse serum. Two-electrode voltage-clamp (Axoclamp 900A amplifier; Axon Instruments) were performed at room temperature (~23°C) in solution containing (in mM) 96 NaCl, 2 KCl, 1 $MgCl_2$, 0.1 $CaCl_2$, 5 HEPES; pH 7.4 with NaOH. Borosilicate glass microelectrodes had resistances of 0.2–0.8 MΩ when backfilled with 3M KCl. Data were digitised at 5 kHz and filtered at 0.5 kHz using pCLAMP 11 software (Digidata 1550B; Axon Instruments). Oocytes were held at −60 mV for all experiments, and solutions applied via gravity fed perfusion into the ~40 µL recording chamber.

Xenopus laevis oocyte surgeries were performed in accordance with the guidelines from the Australian code of practice for the care and use of animals for scientific purposes, 8th edition, 2013. The protocol was approved by the Anatomical Biosciences group of the Animal Ethics Committee at The University of Queensland (Approval Numbers: QBI/AIBN/087/16/NHMRC/ARC, 2018/AE000421, and 2019/AE000097).

2. Supporting Figures

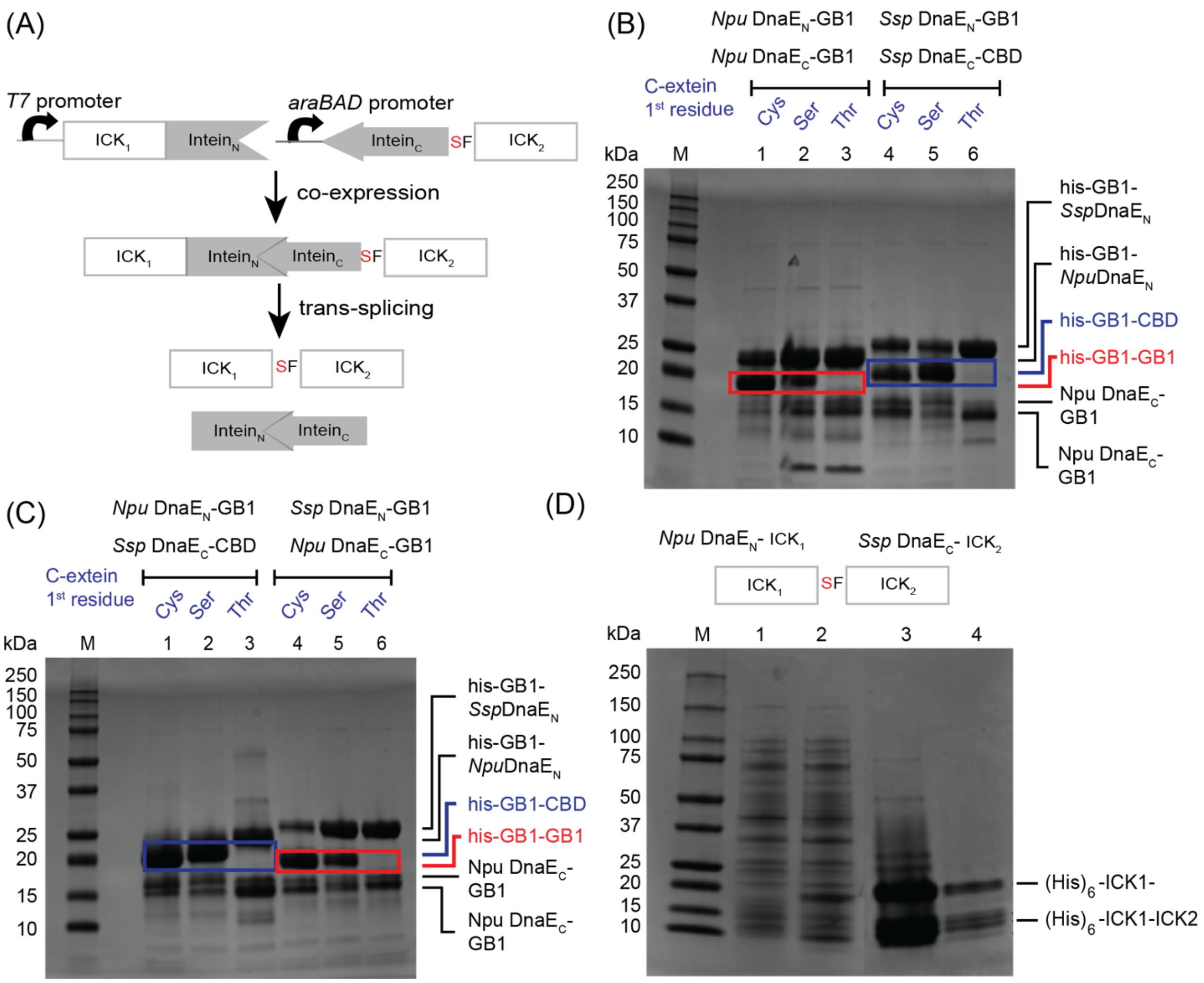

Figure S1. Transplicing efficiency of *Nostoc punctiforme* (Npu) and *Synechocystis sp.* (Ssp) inteins. (A) Schematic drawing of the protein constructs and their ligation. The linker residues are shown in single letter amino acid code. The letter in red is the first residue of C-extein and has been mutated from a native cysteine to a serine or threonine residue for producing disulfide-rich peptides. (B) SDS-PAGE of gene products after *in vivo* protein splicing using split intein systems from Npu DnaE (Lane 1-3) and Ssp DnaE (Lane 4-6). (C) *In vivo* protein splicing using mixed split intein systems. Lane 1-3: combination of *Npu* DnaE$_N$ and *Ssp* DnaE$_C$. Lane 4-6: combination of *Ssp* DnaE$_N$ and *Npu* DnaE$_C$. (D) *In vivo* protein splicing of ICKs of DkTx by *Npu* DnaE$_N$ and *Ssp* DnaE$_C$ split intein systems. Lane 1, before induction; Lane 2, induction with IPTG and then arabinose for co-expression; Lane 3, elution from IMAC; Lane 4, diluted IMAC elution showing the precursor proteins and the *in vivo* ligated DkTx.

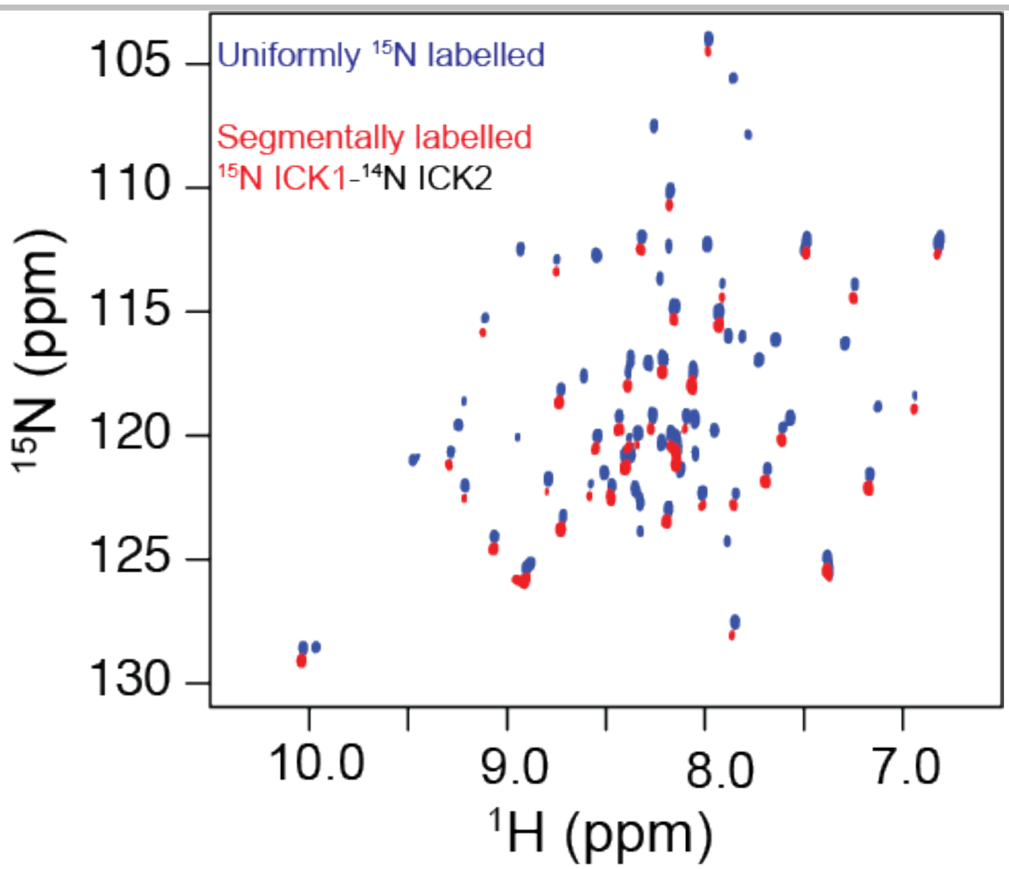

Figure S2. Overlay ^{1}H-^{15}N HSQC spectra of uniformly labelled DkTx (blue) with segmentally labelled DkTx (red), where only ICK1 is isotopically labelled. Note that the spectrum of the uniformly labelled DkTx (blue) has been shifted by 0.5 ppm vertically to aid comparison.

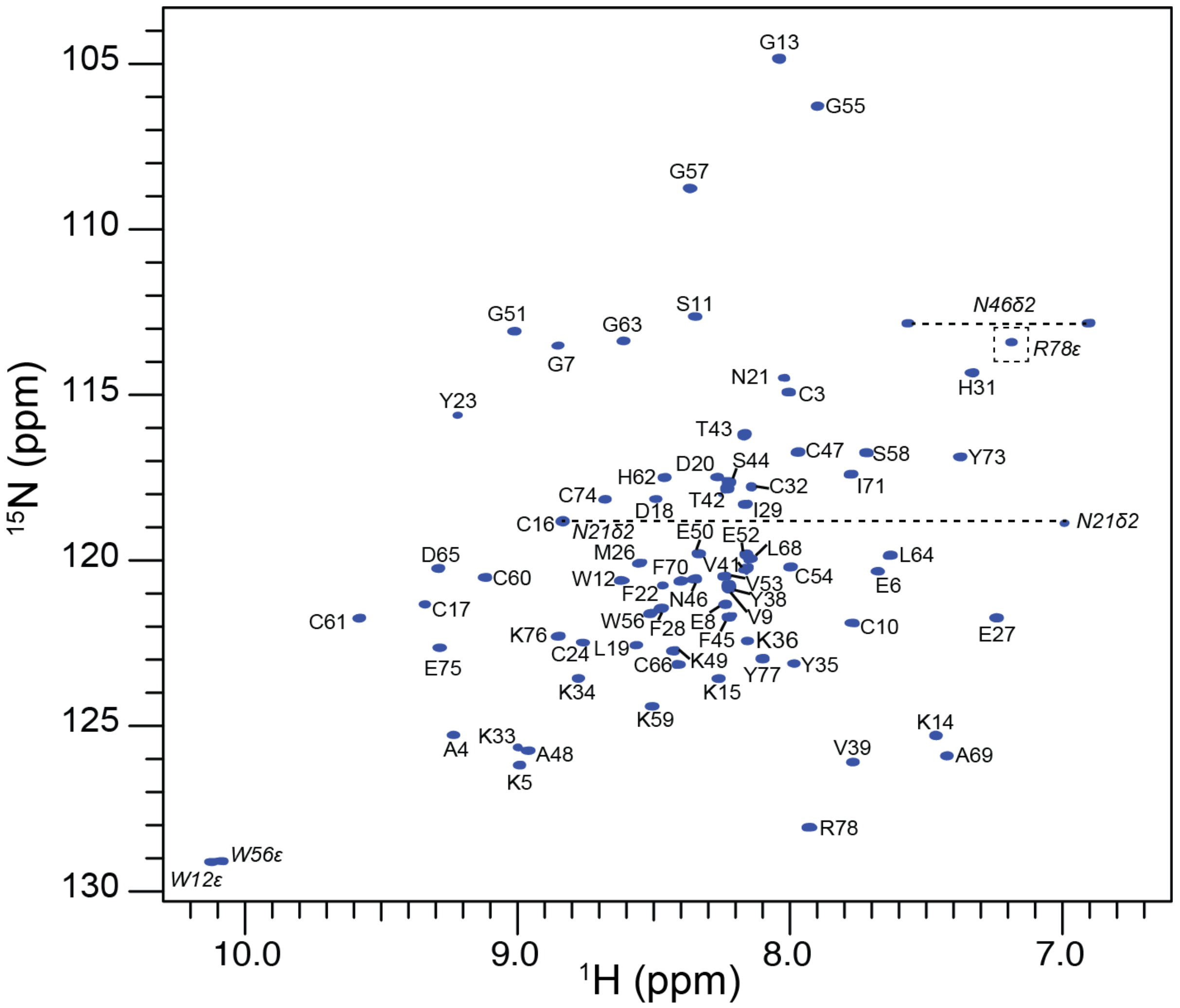

Figure S3. ^1H-^{15}N HSQC spectrum of iDkTx. Peaks represent backbone amide NH correlations. Sidechain correlations are annotated with italic font and the folded arginine sidechains are marked with a dotted box. NH_2 groups are connected using a dashed line.

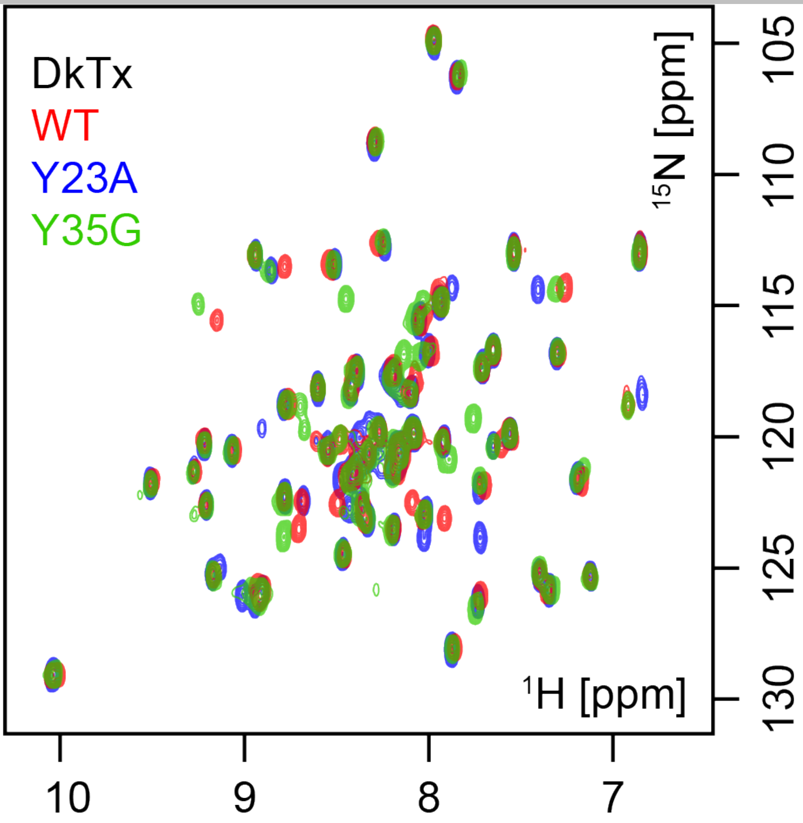

Figure S4. ^{1}H-^{15}N HSQC spectrum of WT DkTx (red) overlayed with Y23A (blue) and Y35G (green) mutants. Overlay shows the peptides to adopt the same fold with similar lineshapes and minor chemical shift changes near the point of mutations (assignment provided in Table S1).

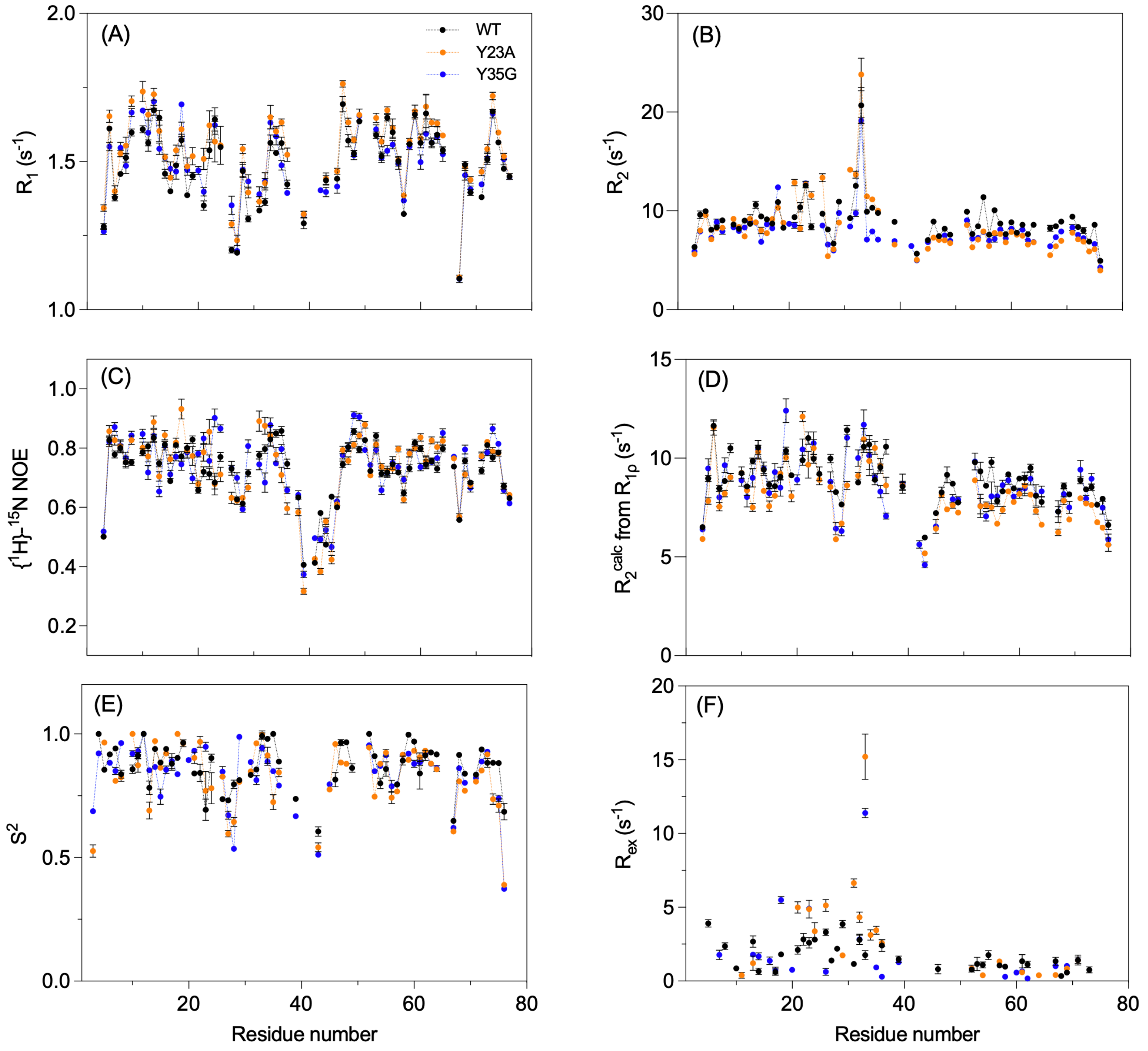

Figure S5. (A) Backbone amide ^{15}N NMR longitudinal (R_1), (B) transverse (R_2) relaxation rate constants, (C) ^{15}N-{1H} steady-state NOE, (D) transverse relaxation rate constants calculated from $R_{1\rho}$, (E) order parameter (S^2) of N-H bond derived from ^{15}N amide relaxation measurements and (F) corresponding exchange rate (R_{ex}) for the all the DkTx variants. These measurements were conducted at a magnetic field strength corresponding to a ^{1}H Larmor frequency of 900 MHz at 298 K.

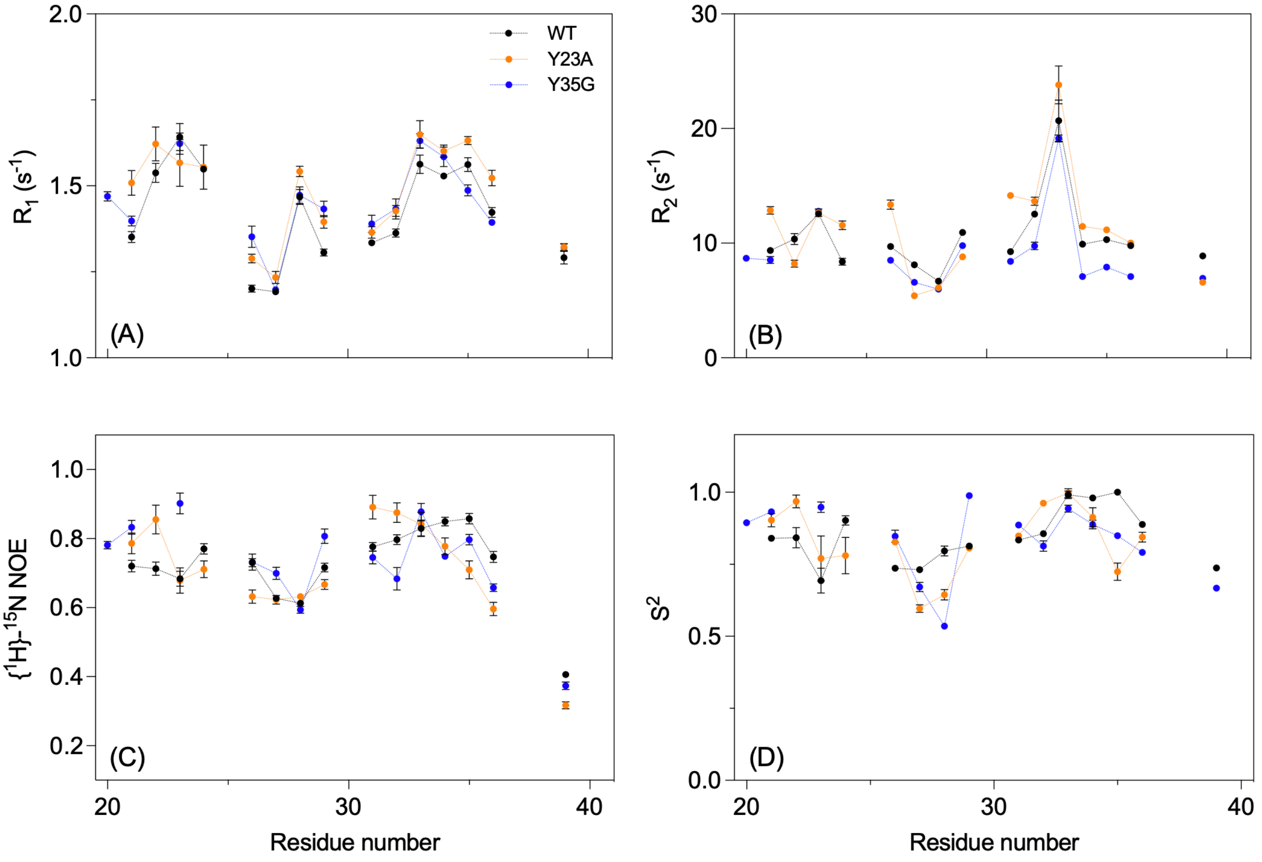

Figure S6. (A-D) Backbone amide ^{15}N longitudinal (R_1) and transverse (R_2) relaxation rate constants, $\{^1H\}$-^{15}N steady-state NOE and order parameter (S^2) for the DkTx variants (residues numbered 20 to 40). These measurements were conducting using a 900 MHz Bruker Avance spectrometer equipped with z-gradient cryoprobe. The data were acquired at 298 K with a <100 μM ^{15}N labeled sample.

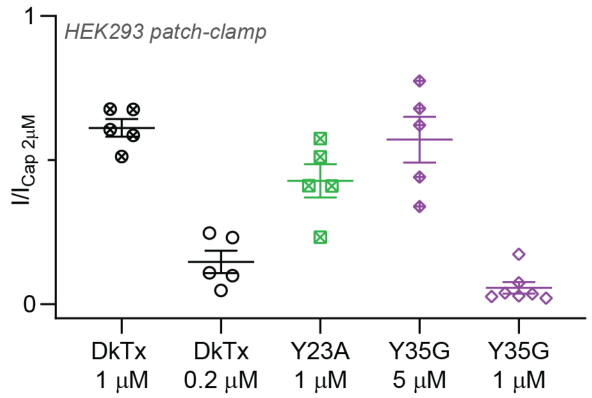

Figure S7. Activity of DkTx and mutants in whole-cell patch-clamp recordings of HEK293 cells expressing human TRPV1. Data represents the ratio between the indicated peptide/concentration and 2 mM capsaicin-evoked current amplitudes at -80 mV. Data are mean ± standard error of the mean (n = 5–7).

3. Supporting Tables

Table 1. NMR structure statistics for iDkTx (6CUC).

Experimental restraints		
Inter-proton distance restraints		
Short-range (i-j<=1)	693	
Medium-range (i-j<5)	109	
Long-range (i-j>5)	206	
Dihedral angle restraints	150 (Φ=59, Ψ=24, χ1=27)	
Disulfide-bond restraints	18	
Total number of restraints per residue	15.1	
RMSD from mean coordinate structure (Å)	All atoms	Backbone
ICK-1: Residues 2-34 (Heavy atoms)	0.84	0.35
ICK-2: Residues 47-77 (Heavy atoms)	0.79	0.18
Stereochemical quality (reported from Molprobity)		
Residues in most favoured Ramachandran region (%)	95.45	
Ramachandran outliers (%)	0	
Unfavourable sidechain rotamers (%)	7.35	
Clashscore, all atoms	5.18	
Overall Molprobity score	2.25 62nd percentile)	

Table S2. Chemical shift assignments of DkTx and Y23A and Y35G mutants.

	WT		Y23A		Y35G	
	δ ^{15}N (ppm)	δ ^{1}H (ppm)	δ ^{15}N (ppm)	δ ^{1}H (ppm)	δ ^{15}N (ppm)	δ ^{1}H (ppm)
**C3**	114.933	8.000	114.969	8.011	114.920	7.997
**A4**	125.319	9.235	125.311	9.247	125.308	9.238
**K5**	126.227	8.989	126.338	9.015	126.297	8.988
**E6**	120.366	7.676	120.416	7.724	120.469	7.719
**G7**	113.550	8.852	113.688	8.922	113.728	8.935
**E8**	121.361	8.234	121.306	8.268	121.422	8.263
**V9**	119.981	8.116	119.973	8.129	119.936	8.129
**C10**	121.929	7.770	122.009	7.798	121.738	7.795
**S11**	112.685	8.344	112.793	8.308	112.657	8.317
**W12**	120.662	8.619	120.553	8.619	120.622	8.611
**G13**	104.916	8.041	105.039	8.044	104.964	8.039
**K14**	125.308	7.466	125.317	7.470	125.148	7.469
**K15**	123.620	8.262	123.611	8.258	123.604	8.269
**C16**	118.855	8.834	118.843	8.857	118.856	8.844
**C17**	121.370	9.336	121.424	9.351	121.435	9.347
**D18**	118.181	8.494	118.346	8.496	118.455	8.505
**L19**	122.590	8.565	122.732	8.501	122.276	8.448
**D20**	117.565	8.275	117.772	8.263	116.941	8.205
**N21**	114.566	8.022	114.363	7.945	114.933	8.102
**F22**	120.853	8.463	119.555	8.393	120.297	8.262
**Y23**	115.648	9.217	119.704	8.977	114.952	9.317
**C24**	122.522	8.760	122.514	8.750	122.551	8.851
**M26**	120.125	8.555	120.091	8.447	120.150	8.555
**E27**	121.739	7.240	121.644	7.264	121.657	7.262
**F28**	121.535	8.483	121.452	8.476	121.442	8.457
**I29**	118.367	8.166	118.424	8.166	118.420	8.166
**H31**	114.364	7.333	114.484	7.478	114.391	7.379
**C32**	117.819	8.140	118.310	8.228	118.360	8.201
**K33**	125.730	8.997	125.014	9.200	125.962	9.026
**K34**	123.578	8.776	126.125	9.082	123.889	8.857
**Y35**	123.138	7.984	123.819	8.096	114.811	8.519
**K36**	122.539	8.161	123.902	7.795	119.370	7.828
**Y38**	120.900	8.218	121.021	8.234	120.912	7.953
**V39**	126.127	7.790	126.360	7.805	126.653	7.819
**V41**	120.565	8.215	120.641	8.223	120.759	8.224
**T42**	117.522	8.264	117.646	8.275	117.791	8.250

**T43**	115.653	8.111	115.635	8.121	115.580	8.142
**N44**	120.747	8.221	120.715	8.213	120.715	8.213
**C45**	116.801	8.061	116.790	8.074	117.019	8.119
**A46**	125.914	8.963	125.958	8.971	126.017	8.978
**K47**	122.735	8.427	122.739	8.434	122.748	8.433
**E48**	119.853	8.338	119.863	8.339	119.865	8.337
**G49**	113.122	9.011	113.118	9.008	113.113	9.009
**E50**	119.877	8.160	119.824	8.160	119.821	8.159
**V51**	120.506	8.240	120.613	8.242	120.601	8.243
**C52**	120.228	7.992	120.217	7.989	120.311	7.996
**G53**	106.344	7.916	106.388	7.919	106.218	7.900
**W54**	121.700	8.519	121.790	8.554	121.706	8.499
**G55**	108.807	8.365	108.940	8.367	108.846	8.355
**S56**	116.784	7.719	116.779	7.723	116.706	7.720
**K57**	124.570	8.528	124.544	8.537	124.492	8.538
**C58**	120.600	9.122	120.622	9.142	120.557	9.138
**C59**	121.808	9.573	121.851	9.580	121.859	9.581
**H60**	117.487	8.476	117.588	8.456	117.571	8.460
**G61**	113.484	8.613	113.486	8.576	113.469	8.588
**L62**	119.924	7.631	119.942	7.630	119.935	7.627
**D63**	120.255	9.288	120.405	9.284	120.368	9.283
**C64**	123.233	8.418	123.270	8.399	123.256	8.403
**L66**	119.947	8.148	120.016	8.148	119.937	8.145
**A67**	125.925	7.424	125.912	7.416	125.915	7.405
**F68**	120.660	8.401	120.739	8.396	120.715	8.393
**I69**	117.469	7.778	117.429	7.780	117.411	7.784
**Y71**	116.909	7.368	116.900	7.377	116.804	7.371
**C72**	118.192	8.676	118.195	8.674	118.196	8.669
**E73**	122.668	9.282	122.696	9.278	122.690	9.275
**K74**	122.367	8.856	122.329	8.853	122.384	8.854
**Y75**	123.052	8.100	123.012	8.084	123.028	8.093
**R76**	128.090	7.932	128.167	7.949	128.154	7.943

Table S3. Diffusion tensor parameters for the prolate model fit to the ^{15}N relaxation data for all the DkTx variants using quadric diffusion (quad_diff) and r2r1 diffusion (r2r1_diff).

		WT			Y23A			Y35G	
		quad_diff	r2r1_diff		quad_diff	r2r1_diff		quad_diff	r2r1_diff
ICK1	τ_C (ns)	4.847	4.871		4.609	4.541		4.418	4.386
	D_{par}/D_{per}	1.240	1.290		1.628	1.694		1.431	1.454
	Theta (°)	1.571	1.565		1.024	1.025		1.646	1.503
	Phi (°)	0.175	0.274		2.052	2.068		6.166	3.019
	X^2_{red}	2.647	2.095		2.449	2.497		0.798	0.775
ICK2	τ_C (ns)	4.481	4.475		3.857	3.843		4.112	4.099
	D_{par}/D_{per}	0.843	0.842		1.255	1.264		0.743	0.755
	Theta (°)	0.650	0.631		1.223	1.239		0.497	0.503
	Phi (°)	5.984	6.016		4.374	4.369		6.342	6.342
	X^2_{red}	1.780	1.838		1.573	1.564		1.654	1.776

Table S4. Washout of DkTx and Y35G evoked currents (I/I_{max}) in *Xenopus* oocytes expressing rat TrpV1 after 15 minutes and HEK293 cells expressing human TRPV1 after 5 minutes. Data are mean ± standard error of the mean. Due to the slow reversibility of some samples, t_{off} measurements were not fit and calculated.

	Oocytes	HEK293
DkTx 1 μM	0.85 ± 0.02	0.76 ± 0.07
DkTx 0.2 μM	0.50 ± 0.11	0.56 ± 0.09
Y35G 5 μM	0.71 ± 0.05	0.47 ± 0.08
Y35G 1 μM	0.20 ± 0.02	0.06 ± 0.02

4. Supporting References

[1] aH. Iwai, S. Zuger, J. Jin, P. H. Tam, *FEBS Lett* **2006**, *580*, 1853-1858; bS. Züger, H. Iwai, *Nat Biotechnol* **2005**, *23*, 736-740.
[2] J. Lobstein, C. A. Emrich, C. Jeans, M. Faulkner, P. Riggs, M. Berkmen, *Microb. Cell Fact.* **2012**, *11*, 56.
[3] C. J. Bohlen, A. Priel, S. Zhou, D. King, J. Siemens, D. Julius, *Cell* **2010**, *141*, 834-845.
[4] J. K. Klint, S. Senff, N. J. Saez, R. Seshadri, H. Y. Lau, N. S. Bende, E. A. Undheim, L. D. Rash, M. Mobli, G. F. King, *PLoS One* **2013**, *8*, e63865.
[5] C. Bae, J. Kalia, I. Song, J. Yu, H. H. Kim, K. J. Swartz, J. I. Kim, *PLoS One* **2012**, *7*, e51516.
[6] M. Mobli, *J. Magn. Reson.* **2015**, *256*, 60-69.
[7] S. Grzesiek, A. Bax, *J. Magn. Reson.* **1992**, *96*, 432-440.
[8] S. Grzesiek, A. Bax, *J. Biomol. NMR* **1993**, *3*, 185-204.
[9] M. Wittekind, L. Mueller, *J. Magn. Reson.* **1993**, *101*, 201-205.
[10] L. E. Kay, G. Xu, A. Singer, D. R. Muhandiram, J. D. Formankay, *J. Magn. Reson.* **1993**, *101*, 333-337.
[11] A. L. Davis, J. Keeler, E. D. Laue, D. Moskau, *J. Magn. Reson.* **1992**, *98*, 207-216.
[12] L. E. Kay, P. Keifer, T. Saarinen, *J. Am. Chem. Soc.* **1992**, *114*, 10663-10665.
[13] T. Miljenović, X. Jia, P. Lavrencic, B. Kobe, M. Mobli, *Journal of Biomolecular NMR* **2017**, *68*, 119-127.
[14] J. C. Hoch, A. S. Stern, *NMR data processing*, Wiley-Liss New York:, **1996**.
[15] W. F. Vranken, W. Boucher, T. J. Stevens, R. H. Fogh, A. Pajon, M. Llinas, E. L. Ulrich, J. L. Markley, J. Ionides, E. D. Laue, *Proteins* **2005**, *59*, 687-696.

Author Contributions

VR, TC, BC, JRD, XJ, MM, SJ, LM, IV and MM performed the experiments and analysed the data. VR and MM produced the original drafts. VR, TC, BC and MM produced the final drafts. All authors contributed to the writing of the final version. MM and IV administered the project.
